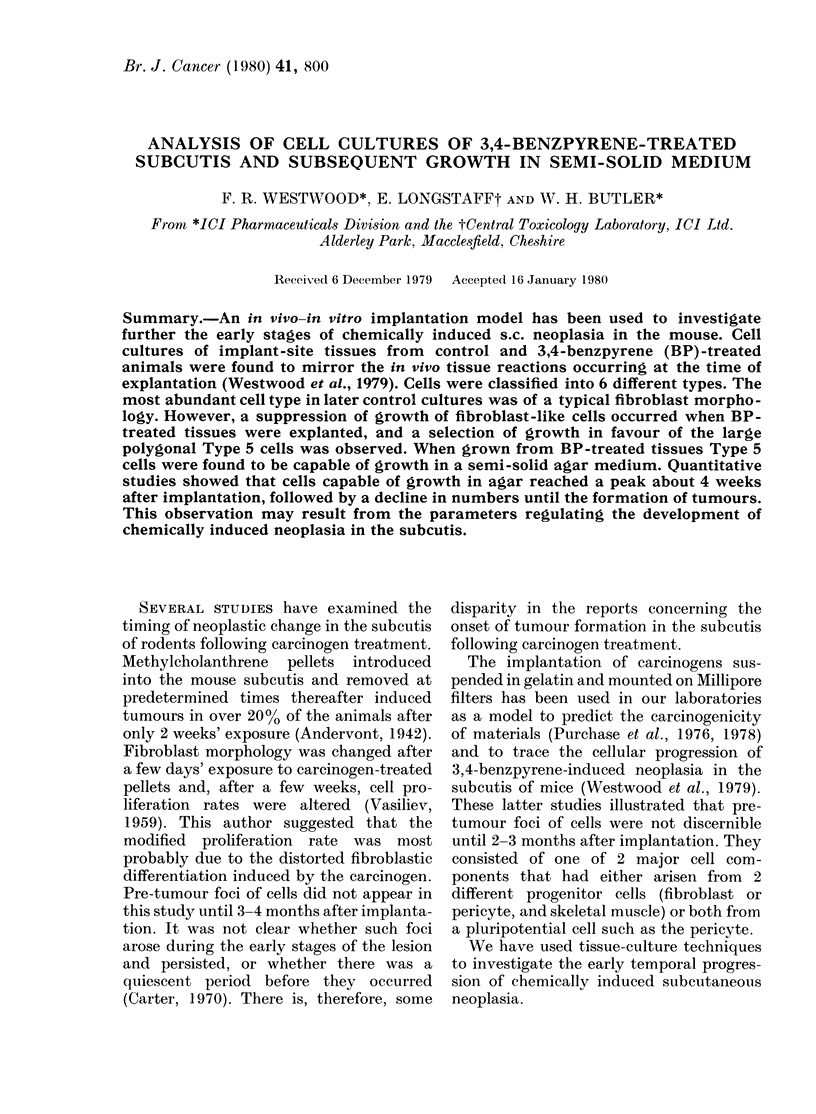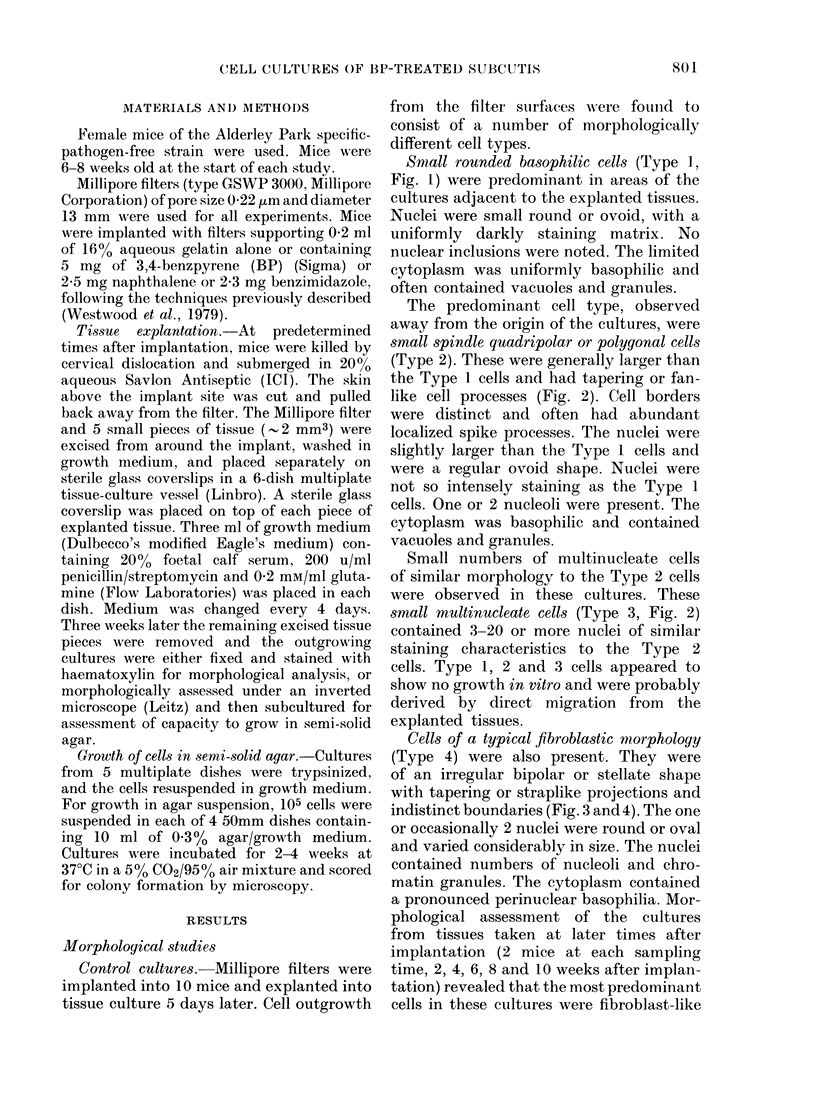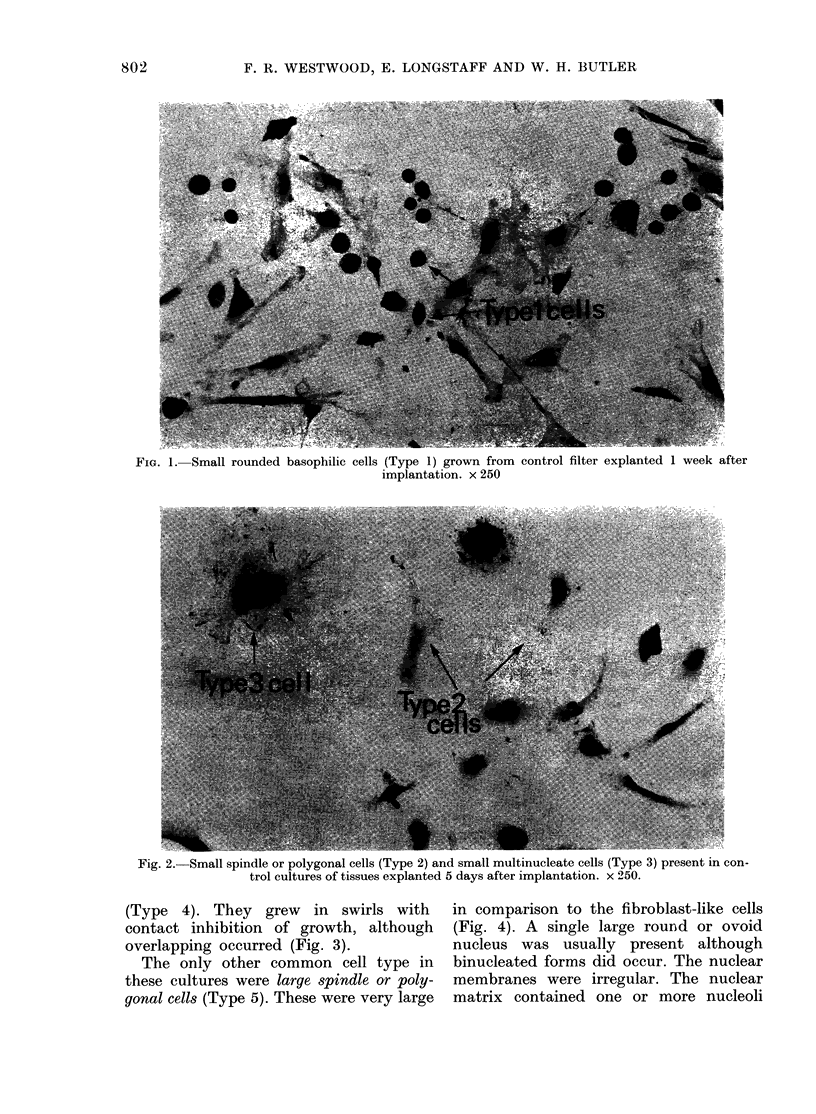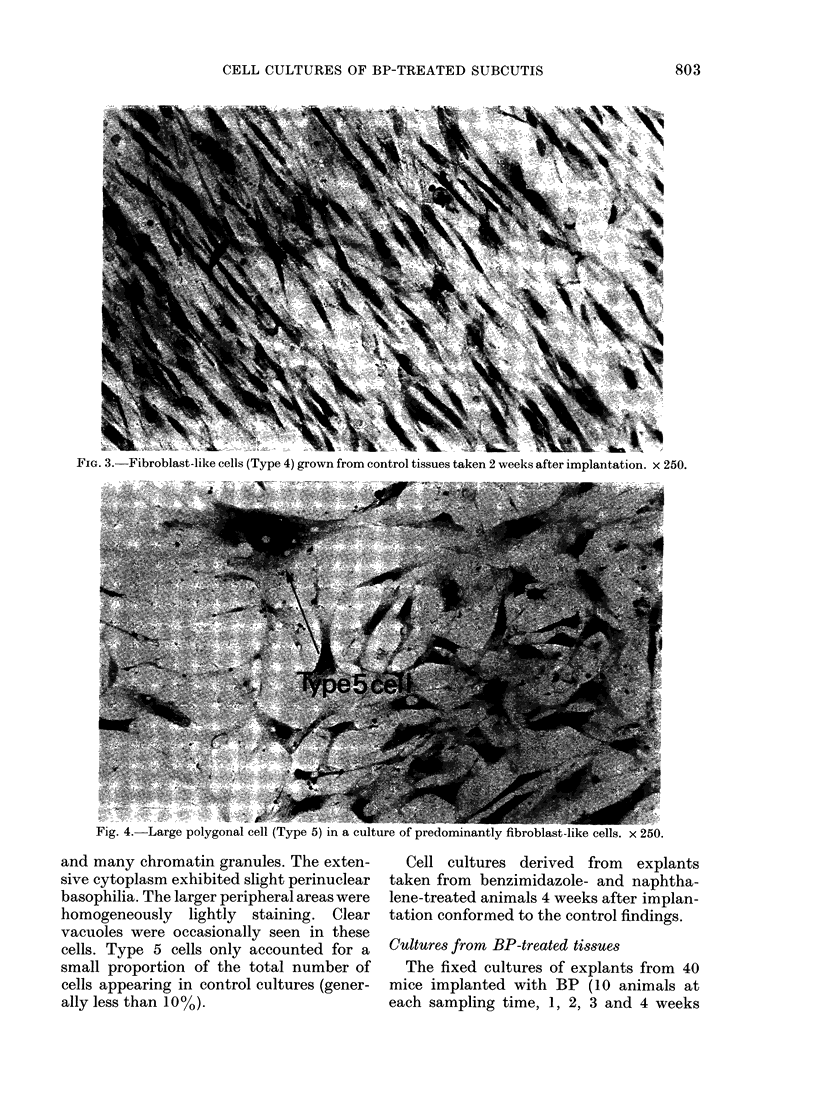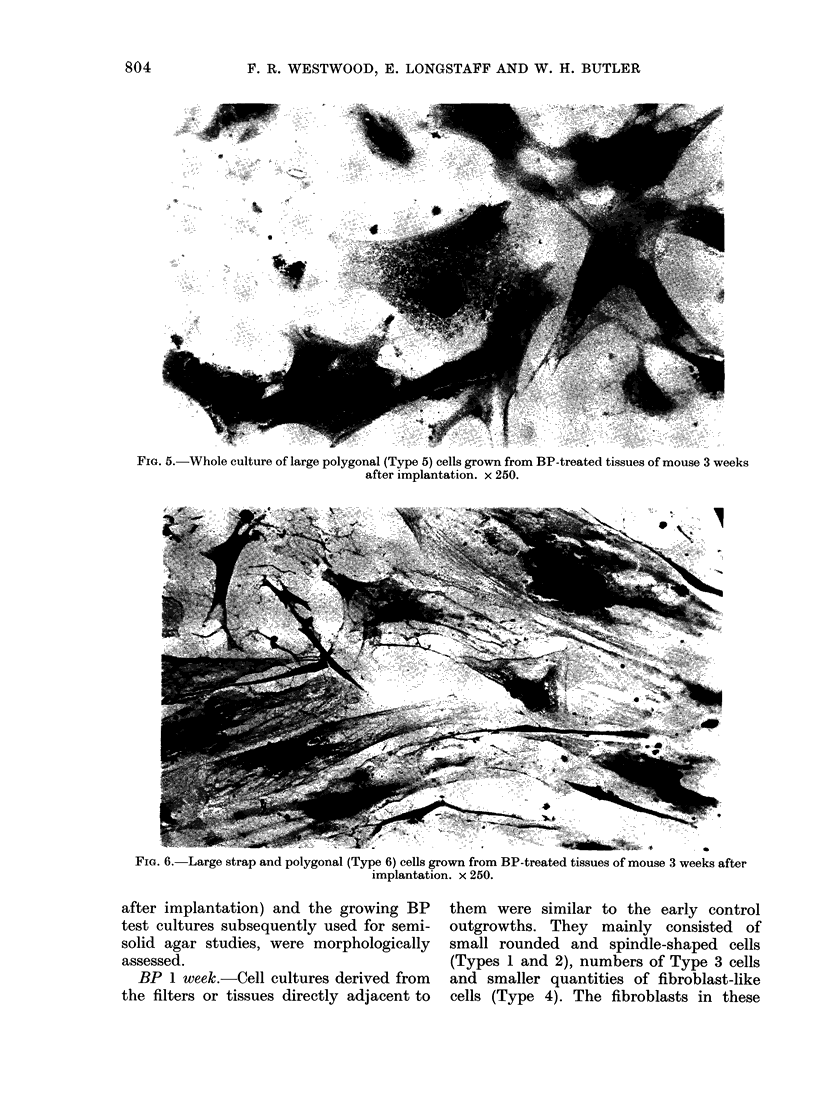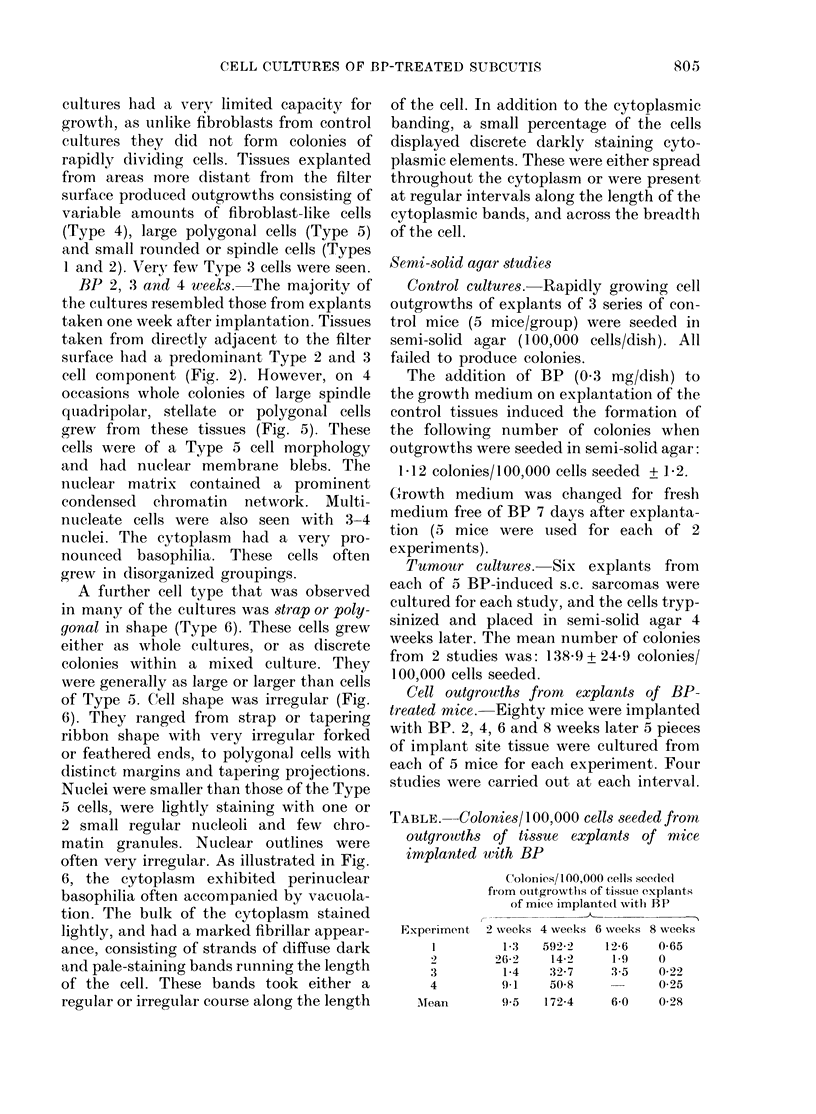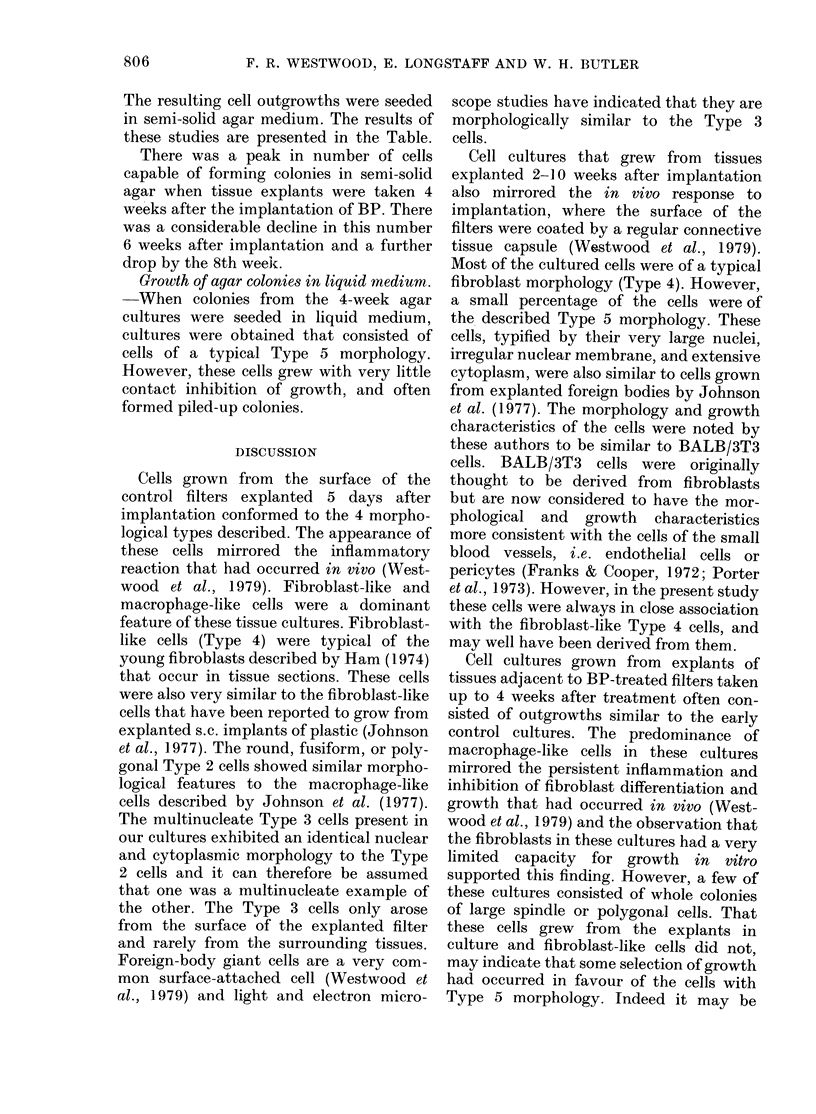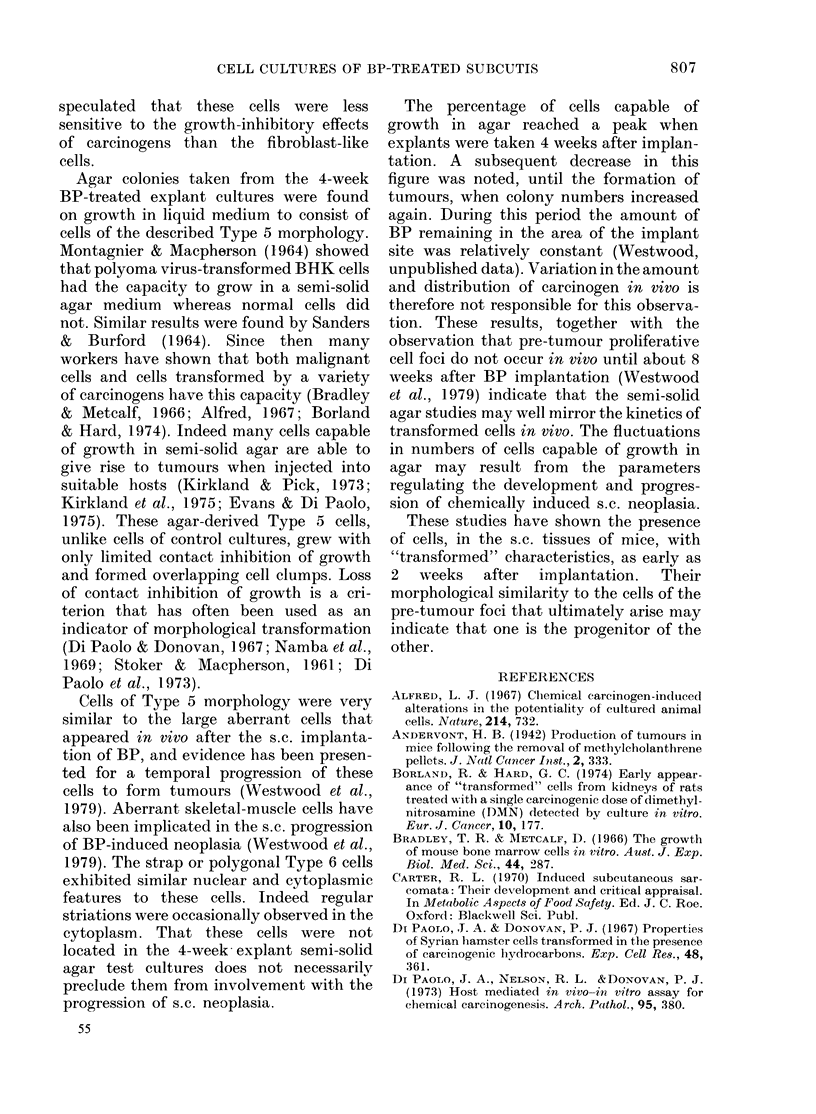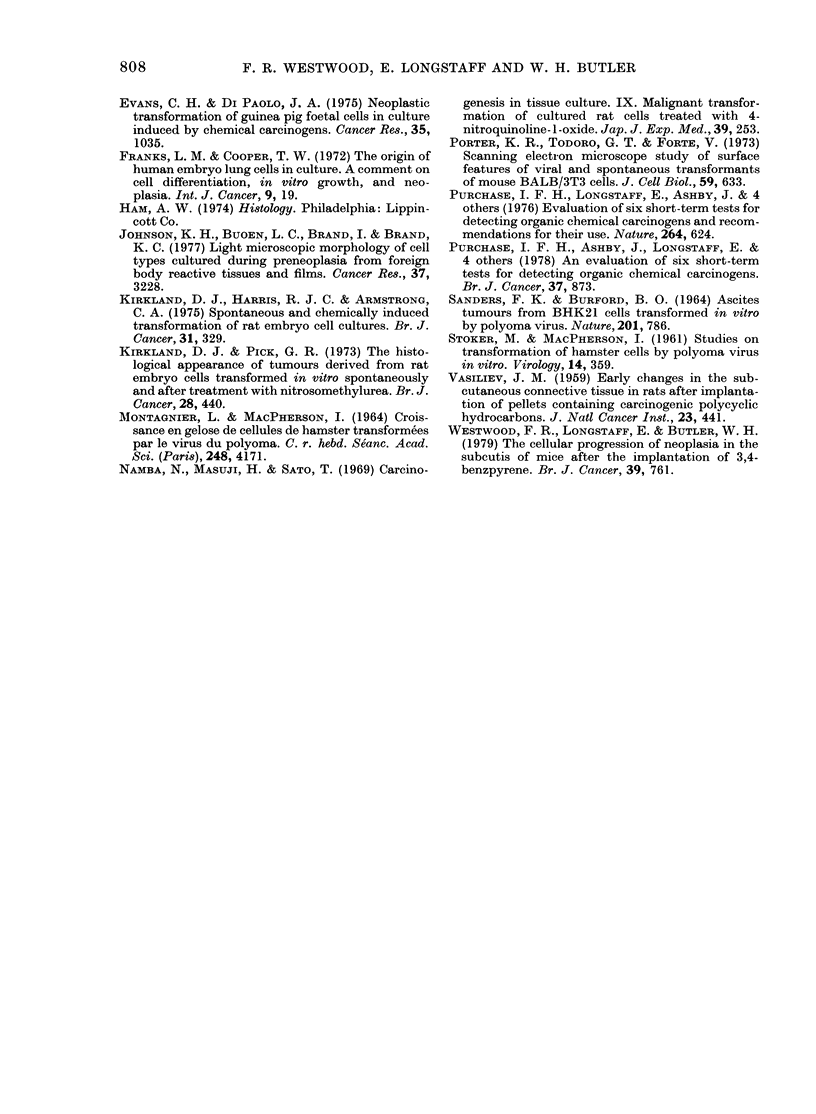# Analysis of cell cultures of 3,4-benzpyrene-treated subcutis and subsequent growth in semi-solid medium.

**DOI:** 10.1038/bjc.1980.144

**Published:** 1980-05

**Authors:** F. R. Westwood, E. Longstaff, W. H. Butler

## Abstract

**Images:**


*Br. J. Cancer* (1980) **41**, 800

### ANALYSIS OF CELL CULTURES OF 3,4-BENZPYRENE-TREATED SUBCUTIS AND SUBSEQUENT GROWTH IN SEMI-SOLID MEDIUM

F. R. WESTWOOD*, E. LONGSTAFF† AND W. H. BUTLER*

*From *ICI Pharmaceuticals Division and the †Central Toxicology Laboratory, ICI Ltd. Alderley Park, Macclesfield, Cheshire*

Received 6 December 1979 Accepted 16 January 1980

**Summary.—An *in vivo–in vitro* implantation model has been used to investigate further the early stages of chemically induced s.c. neoplasia in the mouse. Cell cultures of implant-site tissues from control and 3,4-benzpyrene (BP)-treated animals were found to mirror the *in vivo* tissue reactions occurring at the time of explantation (Westwood *et al.*, 1979). Cells were classified into 6 different types. The most abundant cell type in later control cultures was of a typical fibroblast morphology. However, a suppression of growth of fibroblast-like cells occurred when BP-treated tissues were explanted, and a selection of growth in favour of the large polygonal Type 5 cells was observed. When grown from BP-treated tissues Type 5 cells were found to be capable of growth in a semi-solid agar medium. Quantitative studies showed that cells capable of growth in agar reached a peak about 4 weeks after implantation, followed by a decline in numbers until the formation of tumours. This observation may result from the parameters regulating the development of chemically induced neoplasia in the subcutis.**

SEVERAL STUDIES have examined the timing of neoplastic change in the subcutis of rodents following carcinogen treatment. Methylcholanthrene pellets introduced into the mouse subcutis and removed at predetermined times thereafter induced tumours in over 20% of the animals after only 2 weeks' exposure (Andervont, 1942). Fibroblast morphology was changed after a few days' exposure to carcinogen-treated pellets and, after a few weeks, cell proliferation rates were altered (Vasiliev, 1959). This author suggested that the modified proliferation rate was most probably due to the distorted fibroblastic differentiation induced by the carcinogen. Pre-tumour foci of cells did not appear in this study until 3–4 months after implantation. It was not clear whether such foci arose during the early stages of the lesion and persisted, or whether there was a quiescent period before they occurred (Carter, 1970). There is, therefore, some disparity in the reports concerning the onset of tumour formation in the subcutis following carcinogen treatment.

The implantation of carcinogens suspended in gelatin and mounted on Millipore filters has been used in our laboratories as a model to predict the carcinogenicity of materials (Purchase *et al.*, 1976, 1978) and to trace the cellular progression of 3,4-benzpyrene-induced neoplasia in the subcutis of mice (Westwood *et al.*, 1979). These latter studies illustrated that pre-tumour foci of cells were not discernible until 2–3 months after implantation. They consisted of one of 2 major cell components that had either arisen from 2 different progenitor cells (fibroblast or pericyte, and skeletal muscle) or both from a pluripotential cell such as the pericyte.

We have used tissue-culture techniques to investigate the early temporal progression of chemically induced subcutaneous neoplasia.

### MATERIALS AND METHODS

Female mice of the Alderley Park specific-pathogen-free strain were used. Mice were 6–8 weeks old at the start of each study.

Millipore filters (type GSWP 3000, Millipore Corporation) of pore size 0·22 $\mu$m and diameter 13 mm were used for all experiments. Mice were implanted with filters supporting 0·2 ml of 16% aqueous gelatin alone or containing 5 mg of 3,4-benzpyrene (BP) (Sigma) or 2·5 mg naphthalene or 2·3 mg benzimidazole, following the techniques previously described (Westwood *et al.*, 1979).

*Tissue explantation.*—At predetermined times after implantation, mice were killed by cervical dislocation and submerged in 20% aqueous Savlon Antiseptic (ICI). The skin above the implant site was cut and pulled back away from the filter. The Millipore filter and 5 small pieces of tissue ($\sim$2 mm$^3$) were excised from around the implant, washed in growth medium, and placed separately on sterile glass coverslips in a 6-dish multiplate tissue-culture vessel (Linbro). A sterile glass coverslip was placed on top of each piece of explanted tissue. Three ml of growth medium (Dulbecco's modified Eagle's medium) containing 20% foetal calf serum, 200 u/ml penicillin/streptomycin and 0·2 mM/ml glutamine (Flow Laboratories) was placed in each dish. Medium was changed every 4 days. Three weeks later the remaining excised tissue pieces were removed and the outgrowing cultures were either fixed and stained with haematoxylin for morphological analysis, or morphologically assessed under an inverted microscope (Leitz) and then subcultured for assessment of capacity to grow in semi-solid agar.

*Growth of cells in semi-solid agar.*—Cultures from 5 multiplate dishes were trypsinized, and the cells resuspended in growth medium. For growth in agar suspension, $10^5$ cells were suspended in each of 4 50mm dishes containing 10 ml of 0·3% agar/growth medium. Cultures were incubated for 2–4 weeks at 37°C in a 5% $CO_2$/95% air mixture and scored for colony formation by microscopy.

### RESULTS

### *Morphological studies*

*Control cultures.*—Millipore filters were implanted into 10 mice and explanted into tissue culture 5 days later. Cell outgrowth from the filter surfaces were found to consist of a number of morphologically different cell types.

*Small rounded basophilic cells* (Type 1, Fig. 1) were predominant in areas of the cultures adjacent to the explanted tissues. Nuclei were small round or ovoid, with a uniformly darkly staining matrix. No nuclear inclusions were noted. The limited cytoplasm was uniformly basophilic and often contained vacuoles and granules.

The predominant cell type, observed away from the origin of the cultures, were *small spindle quadripolar or polygonal cells* (Type 2). These were generally larger than the Type 1 cells and had tapering or fan-like cell processes (Fig. 2). Cell borders were distinct and often had abundant localized spike processes. The nuclei were slightly larger than the Type 1 cells and were a regular ovoid shape. Nuclei were not so intensely staining as the Type 1 cells. One or 2 nucleoli were present. The cytoplasm was basophilic and contained vacuoles and granules.

Small numbers of multinucleate cells of similar morphology to the Type 2 cells were observed in these cultures. These *small multinucleate cells* (Type 3, Fig. 2) contained 3–20 or more nuclei of similar staining characteristics to the Type 2 cells. Type 1, 2 and 3 cells appeared to show no growth *in vitro* and were probably derived by direct migration from the explanted tissues.

*Cells of a typical fibroblastic morphology* (Type 4) were also present. They were of an irregular bipolar or stellate shape with tapering or straplike projections and indistinct boundaries (Fig. 3 and 4). The one or occasionally 2 nuclei were round or oval and varied considerably in size. The nuclei contained numbers of nucleoli and chromatin granules. The cytoplasm contained a pronounced perinuclear basophilia. Morphological assessment of the cultures from tissues taken at later times after implantation (2 mice at each sampling time, 2, 4, 6, 8 and 10 weeks after implantation) revealed that the most predominant cells in these cultures were fibroblast-like

FIG. 1.—Small rounded basophilic cells (Type 1) grown from control filter explanted 1 week after implantation. ×250

Fig. 2.—Small spindle or polygonal cells (Type 2) and small multinucleate cells (Type 3) present in control cultures of tissues explanted 5 days after implantation. ×250.

(Type 4). They grew in swirls with contact inhibition of growth, although overlapping occurred (Fig. 3).

The only other common cell type in these cultures were *large spindle or polygonal cells* (Type 5). These were very large in comparison to the fibroblast-like cells (Fig. 4). A single large round or ovoid nucleus was usually present although binucleated forms did occur. The nuclear membranes were irregular. The nuclear matrix contained one or more nucleoli

FIG. 3.—Fibroblast-like cells (Type 4) grown from control tissues taken 2 weeks after implantation. × 250.

Fig. 4.—Large polygonal cell (Type 5) in a culture of predominantly fibroblast-like cells. × 250.

and many chromatin granules. The extensive cytoplasm exhibited slight perinuclear basophilia. The larger peripheral areas were homogeneously lightly staining. Clear vacuoles were occasionally seen in these cells. Type 5 cells only accounted for a small proportion of the total number of cells appearing in control cultures (generally less than 10%).

Cell cultures derived from explants taken from benzimidazole- and naphthalene-treated animals 4 weeks after implantation conformed to the control findings.

*Cultures from BP-treated tissues*

The fixed cultures of explants from 40 mice implanted with BP (10 animals at each sampling time, 1, 2, 3 and 4 weeks

FIG. 5.—Whole culture of large polygonal (Type 5) cells grown from BP-treated tissues of mouse 3 weeks after implantation. × 250.

FIG. 6.—Large strap and polygonal (Type 6) cells grown from BP-treated tissues of mouse 3 weeks after implantation. × 250.

after implantation) and the growing BP test cultures subsequently used for semi-solid agar studies, were morphologically assessed.

*BP 1 week*.—Cell cultures derived from the filters or tissues directly adjacent to them were similar to the early control outgrowths. They mainly consisted of small rounded and spindle-shaped cells (Types 1 and 2), numbers of Type 3 cells and smaller quantities of fibroblast-like cells (Type 4). The fibroblasts in these cultures had a very limited capacity for growth, as unlike fibroblasts from control cultures they did not form colonies of rapidly dividing cells. Tissues explanted from areas more distant from the filter surface produced outgrowths consisting of variable amounts of fibroblast-like cells (Type 4), large polygonal cells (Type 5) and small rounded or spindle cells (Types 1 and 2). Very few Type 3 cells were seen.

*BP* 2, 3 *and* 4 *weeks*.—The majority of the cultures resembled those from explants taken one week after implantation. Tissues taken from directly adjacent to the filter surface had a predominant Type 2 and 3 cell component (Fig. 2). However, on 4 occasions whole colonies of large spindle quadripolar, stellate or polygonal cells grew from these tissues (Fig. 5). These cells were of a Type 5 cell morphology and had nuclear membrane blebs. The nuclear matrix contained a prominent condensed chromatin network. Multinucleate cells were also seen with 3–4 nuclei. The cytoplasm had a very pronounced basophilia. These cells often grew in disorganized groupings.

A further cell type that was observed in many of the cultures was *strap or polygonal* in shape (Type 6). These cells grew either as whole cultures, or as discrete colonies within a mixed culture. They were generally as large or larger than cells of Type 5. Cell shape was irregular (Fig. 6). They ranged from strap or tapering ribbon shape with very irregular forked or feathered ends, to polygonal cells with distinct margins and tapering projections. Nuclei were smaller than those of the Type 5 cells, were lightly staining with one or 2 small regular nucleoli and few chromatin granules. Nuclear outlines were often very irregular. As illustrated in Fig. 6, the cytoplasm exhibited perinuclear basophilia often accompanied by vacuolation. The bulk of the cytoplasm stained lightly, and had a marked fibrillar appearance, consisting of strands of diffuse dark and pale-staining bands running the length of the cell. These bands took either a regular or irregular course along the length of the cell. In addition to the cytoplasmic banding, a small percentage of the cells displayed discrete darkly staining cytoplasmic elements. These were either spread throughout the cytoplasm or were present at regular intervals along the length of the cytoplasmic bands, and across the breadth of the cell.

*Semi-solid agar studies*

*Control cultures*.—Rapidly growing cell outgrowths of explants of 3 series of control mice (5 mice/group) were seeded in semi-solid agar (100,000 cells/dish). All failed to produce colonies.

The addition of BP (0·3 mg/dish) to the growth medium on explantation of the control tissues induced the formation of the following number of colonies when outgrowths were seeded in semi-solid agar:

1·12 colonies/100,000 cells seeded $\pm$ 1·2.

Growth medium was changed for fresh medium free of BP 7 days after explantation (5 mice were used for each of 2 experiments).

*Tumour cultures*.—Six explants from each of 5 BP-induced s.c. sarcomas were cultured for each study, and the cells trypsinized and placed in semi-solid agar 4 weeks later. The mean number of colonies from 2 studies was: 138·9 $\pm$ 24·9 colonies/100,000 cells seeded.

*Cell outgrowths from explants of BP-treated mice*.—Eighty mice were implanted with BP. 2, 4, 6 and 8 weeks later 5 pieces of implant site tissue were cultured from each of 5 mice for each experiment. Four studies were carried out at each interval.

TABLE.—*Colonies/*100,000 *cells seeded from outgrowths of tissue explants of mice implanted with BP*

| | Colonies/100,000 cells seeded from outgrowths of tissue explants of mice implanted with BP | | | |
|---|---|---|---|---|
| Experiment | 2 weeks | 4 weeks | 6 weeks | 8 weeks |
| 1 | 1·3 | 592·2 | 12·6 | 0·65 |
| 2 | 26·2 | 14·2 | 1·9 | 0 |
| 3 | 1·4 | 32·7 | 3·5 | 0·22 |
| 4 | 9·1 | 50·8 | — | 0·25 |
| Mean | 9·5 | 172·4 | 6·0 | 0·28 |

The resulting cell outgrowths were seeded in semi-solid agar medium. The results of these studies are presented in the Table.

There was a peak in number of cells capable of forming colonies in semi-solid agar when tissue explants were taken 4 weeks after the implantation of BP. There was a considerable decline in this number 6 weeks after implantation and a further drop by the 8th week.

*Growth of agar colonies in liquid medium.*—When colonies from the 4-week agar cultures were seeded in liquid medium, cultures were obtained that consisted of cells of a typical Type 5 morphology. However, these cells grew with very little contact inhibition of growth, and often formed piled-up colonies.

### DISCUSSION

Cells grown from the surface of the control filters explanted 5 days after implantation conformed to the 4 morphological types described. The appearance of these cells mirrored the inflammatory reaction that had occurred *in vivo* (Westwood *et al.*, 1979). Fibroblast-like and macrophage-like cells were a dominant feature of these tissue cultures. Fibroblast-like cells (Type 4) were typical of the young fibroblasts described by Ham (1974) that occur in tissue sections. These cells were also very similar to the fibroblast-like cells that have been reported to grow from explanted s.c. implants of plastic (Johnson *et al.*, 1977). The round, fusiform, or polygonal Type 2 cells showed similar morphological features to the macrophage-like cells described by Johnson *et al.* (1977). The multinucleate Type 3 cells present in our cultures exhibited an identical nuclear and cytoplasmic morphology to the Type 2 cells and it can therefore be assumed that one was a multinucleate example of the other. The Type 3 cells only arose from the surface of the explanted filter and rarely from the surrounding tissues. Foreign-body giant cells are a very common surface-attached cell (Westwood *et al.*, 1979) and light and electron microscope studies have indicated that they are morphologically similar to the Type 3 cells.

Cell cultures that grew from tissues explanted 2–10 weeks after implantation also mirrored the *in vivo* response to implantation, where the surface of the filters were coated by a regular connective tissue capsule (Westwood *et al.*, 1979). Most of the cultured cells were of a typical fibroblast morphology (Type 4). However, a small percentage of the cells were of the described Type 5 morphology. These cells, typified by their very large nuclei, irregular nuclear membrane, and extensive cytoplasm, were also similar to cells grown from explanted foreign bodies by Johnson *et al.* (1977). The morphology and growth characteristics of the cells were noted by these authors to be similar to BALB/3T3 cells. BALB/3T3 cells were originally thought to be derived from fibroblasts but are now considered to have the morphological and growth characteristics more consistent with the cells of the small blood vessels, *i.e.* endothelial cells or pericytes (Franks & Cooper, 1972; Porter *et al.*, 1973). However, in the present study these cells were always in close association with the fibroblast-like Type 4 cells, and may well have been derived from them.

Cell cultures grown from explants of tissues adjacent to BP-treated filters taken up to 4 weeks after treatment often consisted of outgrowths similar to the early control cultures. The predominance of macrophage-like cells in these cultures mirrored the persistent inflammation and inhibition of fibroblast differentiation and growth that had occurred *in vivo* (Westwood *et al.*, 1979) and the observation that the fibroblasts in these cultures had a very limited capacity for growth *in vitro* supported this finding. However, a few of these cultures consisted of whole colonies of large spindle or polygonal cells. That these cells grew from the explants in culture and fibroblast-like cells did not, may indicate that some selection of growth had occurred in favour of the cells with Type 5 morphology. Indeed it may be speculated that these cells were less sensitive to the growth-inhibitory effects of carcinogens than the fibroblast-like cells.

Agar colonies taken from the 4-week BP-treated explant cultures were found on growth in liquid medium to consist of cells of the described Type 5 morphology. Montagnier & Macpherson (1964) showed that polyoma virus-transformed BHK cells had the capacity to grow in a semi-solid agar medium whereas normal cells did not. Similar results were found by Sanders & Burford (1964). Since then many workers have shown that both malignant cells and cells transformed by a variety of carcinogens have this capacity (Bradley & Metcalf, 1966; Alfred, 1967; Borland & Hard, 1974). Indeed many cells capable of growth in semi-solid agar are able to give rise to tumours when injected into suitable hosts (Kirkland & Pick, 1973; Kirkland *et al.*, 1975; Evans & Di Paolo, 1975). These agar-derived Type 5 cells, unlike cells of control cultures, grew with only limited contact inhibition of growth and formed overlapping cell clumps. Loss of contact inhibition of growth is a criterion that has often been used as an indicator of morphological transformation (Di Paolo & Donovan, 1967; Namba *et al.*, 1969; Stoker & Macpherson, 1961; Di Paolo *et al.*, 1973).

Cells of Type 5 morphology were very similar to the large aberrant cells that appeared *in vivo* after the s.c. implantation of BP, and evidence has been presented for a temporal progression of these cells to form tumours (Westwood *et al.*, 1979). Aberrant skeletal-muscle cells have also been implicated in the s.c. progression of BP-induced neoplasia (Westwood *et al.*, 1979). The strap or polygonal Type 6 cells exhibited similar nuclear and cytoplasmic features to these cells. Indeed regular striations were occasionally observed in the cytoplasm. That these cells were not located in the 4-week explant semi-solid agar test cultures does not necessarily preclude them from involvement with the progression of s.c. neoplasia.

The percentage of cells capable of growth in agar reached a peak when explants were taken 4 weeks after implantation. A subsequent decrease in this figure was noted, until the formation of tumours, when colony numbers increased again. During this period the amount of BP remaining in the area of the implant site was relatively constant (Westwood, unpublished data). Variation in the amount and distribution of carcinogen *in vivo* is therefore not responsible for this observation. These results, together with the observation that pre-tumour proliferative cell foci do not occur *in vivo* until about 8 weeks after BP implantation (Westwood *et al.*, 1979) indicate that the semi-solid agar studies may well mirror the kinetics of transformed cells *in vivo*. The fluctuations in numbers of cells capable of growth in agar may result from the parameters regulating the development and progression of chemically induced s.c. neoplasia.

These studies have shown the presence of cells, in the s.c. tissues of mice, with "transformed" characteristics, as early as 2 weeks after implantation. Their morphological similarity to the cells of the pre-tumour foci that ultimately arise may indicate that one is the progenitor of the other.

### REFERENCES

ALFRED, L. J. (1967) Chemical carcinogen-induced alterations in the potentiality of cultured animal cells. *Nature*, **214**, 732.

ANDERVONT, H. B. (1942) Production of tumours in mice following the removal of methylcholanthrene pellets. *J. Natl Cancer Inst.*, **2**, 333.

BORLAND, R. & HARD, G. C. (1974) Early appearance of "transformed" cells from kidneys of rats treated with a single carcinogenic dose of dimethylnitrosamine (DMN) detected by culture *in vitro*. *Eur. J. Cancer*, **10**, 177.

BRADLEY, T. R. & METCALF, D. (1966) The growth of mouse bone marrow cells *in vitro*. *Aust. J. Exp. Biol. Med. Sci.*, **44**, 287.

CARTER, R. L. (1970) Induced subcutaneous sarcomata: Their development and critical appraisal. In *Metabolic Aspects of Food Safety*. Ed. J. C. Roe. Oxford: Blackwell Sci. Publ.

DI PAOLO, J. A. & DONOVAN, P. J. (1967) Properties of Syrian hamster cells transformed in the presence of carcinogenic hydrocarbons. *Exp. Cell Res.*, **48**, 361.

DI PAOLO, J. A., NELSON, R. L. &DONOVAN, P. J. (1973) Host mediated *in vivo–in vitro* assay for chemical carcinogenesis. *Arch. Pathol.*, **95**, 380.

EVANS, C. H. & DI PAOLO, J. A. (1975) Neoplastic transformation of guinea pig foetal cells in culture induced by chemical carcinogens. *Cancer Res.*, **35**, 1035.

FRANKS, L. M. & COOPER, T. W. (1972) The origin of human embryo lung cells in culture. A comment on cell differentiation, *in vitro* growth, and neoplasia. *Int. J. Cancer*, **9**, 19.

HAM, A. W. (1974) *Histology*. Philadelphia: Lippincott Co.

JOHNSON, K. H., BUOEN, L. C., BRAND, I. & BRAND, K. C. (1977) Light microscopic morphology of cell types cultured during preneoplasia from foreign body reactive tissues and films. *Cancer Res.*, **37**, 3228.

KIRKLAND, D. J., HARRIS, R. J. C. & ARMSTRONG, C. A. (1975) Spontaneous and chemically induced transformation of rat embryo cell cultures. *Br. J. Cancer*, **31**, 329.

KIRKLAND, D. J. & PICK, G. R. (1973) The histological appearance of tumours derived from rat embryo cells transformed *in vitro* spontaneously and after treatment with nitrosomethylurea. *Br. J. Cancer*, **28**, 440.

MONTAGNIER, L. & MACPHERSON, I. (1964) Croissance en gelose de cellules de hamster transformées par le virus du polyoma. *C. r. hebd. Séanc. Acad. Sci. (Paris)*, **248**, 4171.

NAMBA, N., MASUJI, H. & SATO, T. (1969) Carcinogenesis in tissue culture. IX. Malignant transformation of cultured rat cells treated with 4-nitroquinoline-1-oxide. *Jap. J. Exp. Med.*, **39**, 253.

PORTER, K. R., TODORO, G. T. & FORTE, V. (1973) Scanning electron microscope study of surface features of viral and spontaneous transformants of mouse BALB/3T3 cells. *J. Cell Biol.*, **59**, 633.

PURCHASE, I. F. H., LONGSTAFF, E., ASHBY, J. & 4 others (1976) Evaluation of six short-term tests for detecting organic chemical carcinogens and recommendations for their use. *Nature*, **264**, 624.

PURCHASE, I. F. H., ASHBY, J., LONGSTAFF, E. & 4 others (1978) An evaluation of six short-term tests for detecting organic chemical carcinogens. *Br. J. Cancer*, **37**, 873.

SANDERS, F. K. & BURFORD, B. O. (1964) Ascites tumours from BHK21 cells transformed *in vitro* by polyoma virus. *Nature*, **201**, 786.

STOKER, M. & MACPHERSON, I. (1961) Studies on transformation of hamster cells by polyoma virus *in vitro*. *Virology*, **14**, 359.

VASILIEV, J. M. (1959) Early changes in the subcutaneous connective tissue in rats after implantation of pellets containing carcinogenic polycyclic hydrocarbons. *J. Natl Cancer Inst.*, **23**, 441.

WESTWOOD, F. R., LONGSTAFF, E. & BUTLER, W. H. (1979) The cellular progression of neoplasia in the subcutis of mice after the implantation of 3,4-benzpyrene. *Br. J. Cancer*, **39**, 761.